# Dynamic auroral storms on Saturn as observed by the Hubble Space Telescope

**DOI:** 10.1002/2014GL060186

**Published:** 2014-05-20

**Authors:** J D Nichols, S V Badman, K H Baines, R H Brown, E J Bunce, J T Clarke, S W H Cowley, F J Crary, M K Dougherty, J-C Gérard, A Grocott, D Grodent, W S Kurth, H Melin, D G Mitchell, W R Pryor, T S Stallard

**Affiliations:** 1 Department of Physics and Astronomy, University of LeicesterLeicester, UK; 2 Department of Physics, Lancaster UniversityLancaster, UK; 3 Space Science and Engineering Center, University of Wisconsin-MadisonMadison, Wisconsin, USA; 4 Lunar and Planetary Lab, University of ArizonaTucson, Arizona, USA; 5 Center for Space Physics, Boston UniversityBoston, Massachusetts, USA; 6 Laboratory for Atmospheric and Space Physics, University of Colorado BoulderBoulder, Colorado, USA; 7 Blackett Laboratory, Imperial College LondonLondon, UK; 8 Laboratoire de Physique Atmospherique et Planetaire, Universite de LiegeLiege, Belgium; 9 Department of Physics and Astronomy, University of IowaIowa City, Iowa, USA; 10 Applied Physics Laboratory, Johns Hopkins UniversityLaurel, Maryland, USA; 11 Department of Science, Central Arizona CollegeCoolidge, Arizona, USA

## Abstract

We present observations of significant dynamics within two UV auroral storms observed on Saturn using the Hubble Space Telescope in April/May 2013. Specifically, we discuss bursts of auroral emission observed at the poleward boundary of a solar wind-induced auroral storm, propagating at ∼330% rigid corotation from near ∼01 h LT toward ∼08 h LT. We suggest that these are indicative of ongoing, bursty reconnection of lobe flux in the magnetotail, providing strong evidence that Saturn's auroral storms are caused by large-scale flux closure. We also discuss the later evolution of a similar storm and show that the emission maps to the trailing region of an energetic neutral atom enhancement. We thus identify the auroral form with the upward field-aligned continuity currents flowing into the associated partial ring current.

## 1. Introduction

Spectacular far ultraviolet (FUV) auroral storms have been previously observed on Saturn using the Hubble Space Telescope (HST), consisting of a considerable brightening and poleward expansion of the aurora on the dawnside, resulting in a factor of ∼2–3 increase in total emitted FUV power [*Prangé et al.*, 2004; *Crary et al.*, 2005; *Clarke et al.*, 2005; *Grodent et al.*, 2005; *Clarke et al.*, 2009]. This morphology has been interpreted theoretically as a manifestation of compression-induced tail reconnection [*Cowley et al.*, 2005], and the effect of IMF direction has also been shown to be a significant factor in controlling the radius of the auroral oval [*Belenkaya et al.*, 2011]. In addition, *Jackman et al.* [2013] related small-scale auroral blobs observed on the nightside using the Cassini Ultraviolet Imaging Spectrograph (UVIS) to field-aligned currents associated with tail reconnection bursts, and recently the latitude of the poleward boundary of the auroral emission has been discussed in terms of unbalanced dayside and nightside reconnection [*Badman et al.*, 2005, 2013]. A somewhat different auroral enhancement was discussed by *Mitchell et al.* [2009], who identified a corotating patch of emission equatorward of the main oval with filamentary field-aligned currents associated with an energetic neutral atom (ENA) enhancement produced by an earlier interval of tail reconnection. However, the dynamics of Saturn's storm time auroral morphology have not heretofore been discussed. In this paper we present HST observations obtained in April/May 2013, which capture significant evolution of the auroral morphology during two auroral storms observed on days 95 and 140, and we identify auroral forms with poleward boundary intensifications and the upward continuity current flowing into the trailing region of a partial ring current, respectively.

## 2. Data

We employ images of Saturn's northern FUV auroral emission obtained by the solar blind channel (SBC) of the Advanced Camera for Surveys (ACS) on board HST over April–May 2013, obtained as part of a long-term program of observations of Saturn's northern pole over the interval 2011–2013. The images were processed using a pipeline that has been extensively discussed previously [see, e.g., *Clarke et al.*, 2009; *Nichols et al.*, 2009], such that here we provide a brief overview. The ACS/SBC detector is a 1024 × 1024 multianode microchannel array, with a field of view of 35 × 31arcsec^2^. We employed the F125LP and F115LP long-pass filters, the former of which admits H_2_ Lyman and Werner bands, while the latter also includes H Lyman-*α*emission. Fifteen orbits were executed over days 95–142 in 2013, and during each orbit nineteen 100 s exposures were obtained. The units were converted from counts to kR (where 1 kR represents a source flux of 10^9^ ph cm^−2^ s^−1^ radiating into 4π steradians) of total H_2_ emission using the conversion factors 1kR = 2.05 × 10^−3^ and 1.20 × 10^−3^ counts *s*^−1^ for the F115LP and F125LP filters, respectively [*Gustin et al.*, 2012], and the images were projected onto a planetocentric latitude-longitude grid assuming an emission altitude of 1100 km [*Gérard et al.*, 2009]. In this letter we concentrate on the data obtained on days 95 and 140, comprising 1 and 3 orbits, respectively, which exhibited morphologies associated with auroral storms.

## 3. Analysis

**Figure 1:**
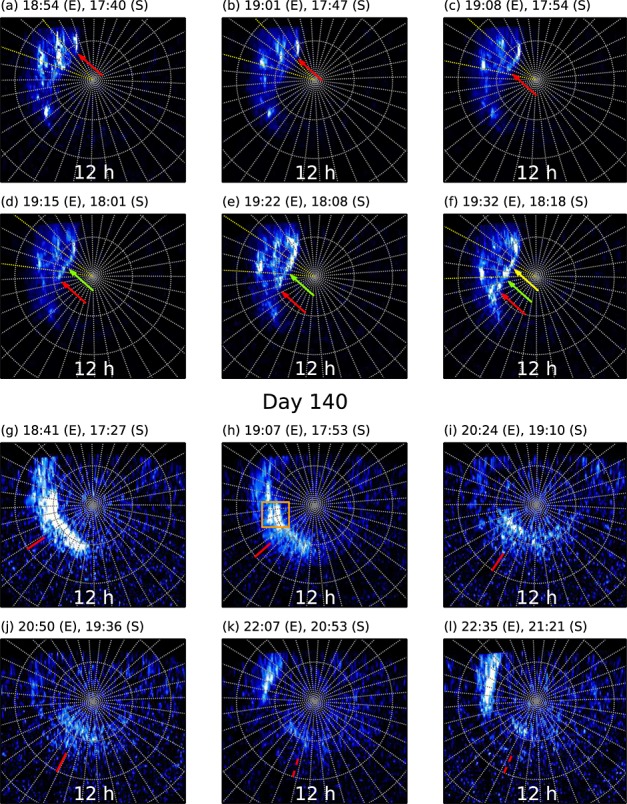
Figure showing images roughly evenly spaced across the orbits which executed on (a–f) day 95 and (g–l) day 140. The images are displayed using a Lambert azimuthal equal-area projection as viewed from above a colatitude of 5° along the central meridian longitude, which is oriented toward the bottom. A grey 10°×10° latitude-longitude grid is overlaid. The labels above each panel indicate the UT of the start of the 100 s exposure at HST (labeled E) and corrected for the one-way light travel time (labelled S). The color scale for Figures 1a–1f is saturated at 80 kR, while for Figures 1g–1l it is log stretched and saturated at 50 kR to highlight fainter emission, as shown by the color bars on the right. In Figures 1a–1f the colored arrows indicate the forward edges of three fast-propagating bursts of emission observed along the poleward edge of the aurora, while the region bounded by the yellow meridians is longitudinally averaged in Figure 2. In Figures 1g–1l the red tick marks indicate the local times of the centers of the Gaussian fits to latitude-integrated intensity profiles, as discussed in the text. Solid lines indicate robust fits, while the dashed lines indicate values for less robust fits. The orange box indicates the double feature discussed in the text.

In Figures 1a–1f we show six example images roughly evenly spaced across the day 95 orbit, and the whole set of images is available as a movie in the supporting information. The morphology is consistent with the previous observations of solar wind-induced storms associated with large-scale tail reconnection events; i.e., the dawnside polar region is mostly filled with bright emission up to high latitudes. The association of the morphology with a solar wind compression event is augmented by a brief excursion of Cassini into the magnetosheath at 16 *R*_*S*_ near to 12 h local time (LT) late on day 94, followed by Cassini Radio and Plasma Wave Science instrument observations of a Saturn kilometric radiation (SKR) low-frequency extension near 06 h on day 95, amid typically enhanced powers over days 94–97. This is consistent with the 8–9 h delay between solar wind compression incidence and 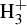 aurora brightening observed by *Stallard et al.* [2012]. In addition, the magnetospheric imaging instrument (MIMI) on board Cassini observed the highest sustained energetic ion intensity at Titan's orbit of any of the previous 49 near-noon (±2 h LT) Titan encounters in the Tour (specifically, the ion intensities below ∼200 keV were a little higher than those for T5, the previous most intense Titan encounter, whereas the intensities above ∼300 keV were about an order of magnitude higher than for T5). Based on previous observations, such intensities are consistent with intense ion heating that almost certainly took place in the post midnight sector, with the heated plasma subsequently rotating through to the dayside [*Mitchell et al.*, 2005, 2009].

**Figure 2:**
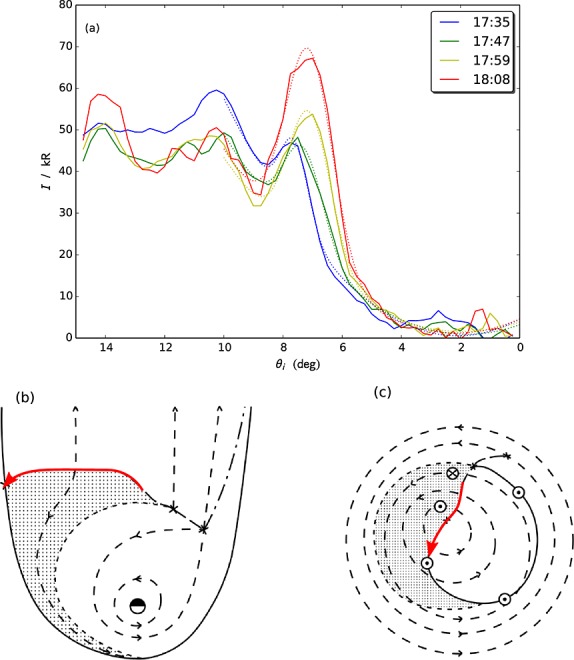
(a) The intensity averaged over five exposures and longitudes 230°–260°, versus colatitude. The legend indicates the light travel-corrected start times of the first image associated with each colored line. The colored dotted lines show the associated Gaussian fits to the peaks at the poleward boundary, as discussed in the text. Figures 2b and 2c show a schematic adapted from *Cowley et al.* [2005] illustrating (b) the equatorial hot plasma and flow streamlines part way through an interval of rapid solar wind-induced tail reconnection and (c) the ionospheric counterpart. A sketch of the path of the day 95 bursts and their equivalent in the equatorial plane are shown by the superimposed red arrows.

The emission on day 95 is patchy, with regions of bright (∼90 kR) aurora superposed on a background of ∼30 kR emission. Initially, a large patch is observed near ∼03 h LT, which gradually fades over the ∼45 min observation interval. Toward the equatorward edge at ∼15°colatitude, the patches generally persist over the entire orbit and subrotate, with the centroids of emission [see, e.g., *Carbary et al.*, 2000] rotating at, e.g., ∼70% and ∼45% of rigid corotation (taken to be relative to the period of the northern SKR oscillations in mid-2013, i.e., ∼10.64 h [*Cowley and Provan*, 2013]) for the patches located near 05 h and 08 h LT, respectively. Particularly, interesting features, however, are three ∼2°-wide bursts of very bright (up to ∼120 kR) emission at the poleward boundary of the aurora at ∼7°colatitude. They originate from ∼01 h LT and quickly propagate roughly along a great circle toward ∼8 h LT, with the head of the surge travelling at a mean speed of ∼4.1 km s^−1^in the ionosphere, i.e., corresponding to ∼330% of rigid corotation (note that the mean angle of the burst propagation to the zonal direction is ∼5°) at 7° colatitude. Three bursts separated by ∼10–15 min are observed during the interval, the forward edges of which, as defined by maxima in the negative of the intensity gradient along the burst direction (bandpass filtered between 50 and 150 pixels to remove noise), are shown by the red, green, and yellow arrows in Figures 1a–1f. We have also coadded groups of five images to increase signal to noise and plotted in Figure 2a the intensity averaged over the 30°-wide longitude sector indicated by the yellow meridians in Figures 1a–1f, versus colatitude. It is apparent that the latitude of the poleward intensity dropoff moves ∼1°poleward from ∼7°to ∼6°colatitude over the course of the orbit. In order to determine the significance of this shift, given the similar ∼1°full width at half maxima of the peaks at the poleward boundary, we have fitted to the region in and around each peak a function comprising a linear combination of a Gaussian and a quadratic “background,” as shown by the dotted lines in Figure 2a. The difference between the best fit poleward HWHM locations for the first (blue) and last (red) profiles is  1°, whereas the uncertainty in this value, given by the RMS of the uncertainties on the Gaussian centers, and HWHMs is ∼0.06°, such that the poleward edges of the peaks are significantly separated. Note that the other auroral features remain essentially static and indeed are not expected to move since the closure (or opening) of open flux does not affect the flux mapping of features on closed field lines—it merely changes the flux shell on which the open-closed field line boundary lies.

**Figure 3:**
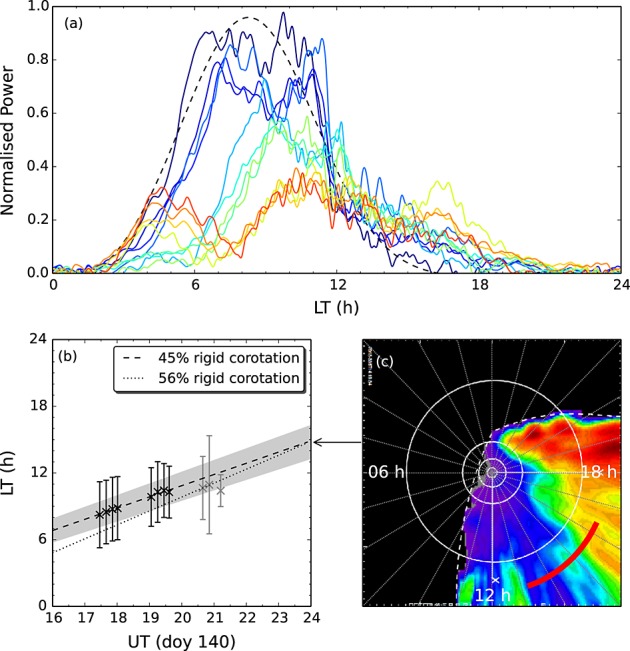
Plot showing the rotational motion of the main auroral form observed on day 140. Figure 3a shows the latitude-integrated intensity, normalized to the maximum observed value, versus local time. The colors denote order of observation, increasing from dark blue to red. The black dashed line indicates the Gaussian plus quadratic function fit to the first image intensity profile. Figure 3b shows the local time of the centers of the Gaussian fits versus UT. The black and grey symbols indicate robust and nonrobust fits, respectively. The grey envelope and black dashed line indicates the linear fit to the robust points along with its uncertainty. The dotted line shows the motion of the double feature discussed in the text. Figure 3c shows the equatorial projection of the 55–90 keV ENA image obtained by Cassini MIMI Ion and Neutral Camera (INCA), with exposure time 23:39:56–23:56:56 UT on day 140. The viewing direction is from ∼03 h LT and ∼45° latitude, and the projection is oriented such that noon is toward the bottom. The extrapolated location of the peak auroral emission is expected to lie along the thick red line. The solid white circles indicate radial distances of 1, 3, 7, and 20 *R*_*S*_, and the dashed white line indicates the INCA field of view.

Considering now the observations on day 140, we similarly show in Figures 1g–1l a selection of images roughly evenly spread across the observing interval, which on this day comprised three contiguous orbits. The emission is characterized initially by a similar, although less pronounced, dawnside poleward expansion, with a thick band of emission in the dawn hemisphere extending over ∼8–15° colatitude, and a particularly bright (up to ∼140 kR) form at the poleward boundary in the morning sector. Over the course of the interval the feature subrotates at ∼45% rigid corotation (see below) and fades at the rate of ∼4.6 GW h^−1^from an initial value of ∼33 GW, and toward the end of the interval a new storm grows in the postmidnight sector. In order to determine the rotation rate of the earlier form, we have first corrected the intensities for limb brightening by multiplication with the cosine of the angle of observation [*Grodent et al.*, 2005] (note, however, that the following results do not sensitively depend on this correction) and integrated the intensities over a colatitude band between 3–18°, with the resulting profiles plotted in Figure 3a. We have then fitted to each profile a function comprising a linear combination of a Gaussian and a quadratic background, as shown by the representative black dashed line in Figure 3a, which indicates the fit to the first image in the sequence, and then plotted the local time of the centers of the Gaussians versus UT in Figure 3b. The black symbols indicate where the Gaussian fit is robust, i.e., where the root-mean-square deviation of the intensity from the fit is significantly less than (<∼10%) the height of the Gaussian, while the grey points indicate lower confidence fits. A weighted linear fit to the centers of the robust Gaussian fits indicates a rotation rate for the auroral feature of ∼45 ± 3% rigid corotation, as mentioned above. This is on the low side of the ∼40–70% of rigid corotation observed for plasma in the outer magnetosphere near 16 *R*_*S*_ by *Thomsen et al.* [2010] but is consistent with the ∼30–60% observed near Titan's orbit by *Arridge et al.* [2011] and is similar to the rotation rate of the patch located in the same LT sector on day 95. The fits plotted in grey possibly indicate a slowing from this reasonably uniform rotation rate, although given the lower confidence of these fits it is unclear whether this slowdown is real. Finally, we have also compared the motion of the whole structure as determined above with that of the prominent double feature indicated by the orange square in Figure 1h. The feature is first located at  6.5 h LT and rotates at  56% rigid corotation, i.e., somewhat faster than the overall rotation rate of the wider form, although it fades throughout the first orbit. We have indicated this motion using the dotted line in Figure 3b.

## 4. Discussion

We now consider the physical origin of the above-discussed auroral forms. Starting with the day 95 event, we first note that the burst speeds are much larger than the flows observed in Saturn's nightside magnetosphere [*McAndrews et al.*, 2009], such that the propagation is likely a phase velocity associated with the field-aligned current system. We also note that these forms bear some resemblance to terrestrial poleward boundary intensifications (PBIs) [*Lyons et al.*, 1999], although we lack the spatial resolution to determine whether they exhibit the same meridional streamer-like morphology of those events. Terrestrial PBIs are associated with ongoing reconnection of lobe flux in the magnetotail, and with this in mind, we refer to the discussion of the auroral response to large-scale compression-induced tail reconnection by *Cowley et al.* [2005]. An extract from their Figure 10 is shown in Figures 2b and 2c, depicting the plasma flow and hot plasma location, and the ionospheric projection of the plasma flow, along with resulting field-aligned currents part way through an interval of rapid closure of open flux. Comparing this schematic with the images in Figures 1a–1f, it is apparent that in this scenario the poleward boundary auroral surges are located in a region of upward field-aligned current induced by the small-scale twin-vortical flow associated with reconnection. It is also worth noting that in Figures 1a–1f, there is no substantial emission near 00 h LT, consistent with the expected downward current in this region. The red arrows in Figure 2c the location and direction of the auroral surges and in Figure 2b the corresponding location and direction in the equatorial plane. We thus suggest that the auroral surges are signatures of ongoing, bursty reconnection of lobe flux in the tail, with reconnection onset propagating rapidly from near midnight to the dawn flank. Over the ∼45 min orbit the first burst propagates entirely across the polar cap. Assuming a tail radius of ∼60 *R*_*S*_ [*Kanani et al.*, 2010], a rough estimate of the speed of reconnection onset propagation across the tail then follows to be ∼1.3*R*_*S*_ min^−1^ or ∼1340km s^−1^. Flux closure is also consistent with the ∼1°poleward motion of the poleward boundary. Modelling the region of closed flux as a 90°×1° annular sector whose outer edge lies on 7°colatitude, along with employing the internal field model of *Burton et al.* [2010], yields an estimate for the open flux change ∼0.75 GWb over the 45 min, corresponding to a reconnection voltage of ∼280 kV. This is consistent with the upper extrema observed by *Badman et al.* [2013] over longer intervals. The auroral morphology during this event is clearly very different from that studied by *Jackman et al.* [2013], who identified small-scale (few degree wide) ∼16–36 kR auroral blobs at lower latitudes on the main oval near midnight with tail dipolarizations observed by Cassini. We suggest that the small-scale forms discussed by *Jackman et al.* [2013] are indicative of steady Vasyliunas or Dungey cycle reconnection, whereas the ∼120 kR cross-polar cap surges reported here are manifestations of rapid, large-scale solar wind compression-induced flux closure events.

Considering now the origin of the auroral feature observed on day 140, we first note that the dawnside poleward expansion is also consistent with a solar wind compression event, and comparison with similar morphologies observed in the south [*Clarke et al.*, 2005; *Grodent et al.*, 2005; *Clarke et al.*, 2009] indicates that the auroras correspond to the later stage of an auroral storm, which probably started out looking more like the day 95 images. In any case, the high latitude of the emission indicates that it maps to the outer magnetosphere. Near to midnight on day 140/141 an ENA image was obtained by MIMI, the equatorial projection of which is shown in Figure 3c. Although there is some radial stretching due to the ∼45°latitude of the spacecraft during the observation, and the relation between ENAs observed at ∼10 *R*_*S*_ and ∼20 *R*_*S*_ is complicated, the local time of the trailing edge of the main enhancement observed here is reasonably apparent, lying at ∼15–16 h LT. Note also that little energy dispersion is observed in the ENAs beyond ∼15 *R*_*S*_ owing to current sheet perturbations [*Birmingham*, 1982]. Assuming that the rotation rate of the auroral emission remained constant at ∼45 ± 3% rigid corotation, and including the uncertainty in the line intercept, extrapolation of the peak location to 24 h UT yields a local time of ∼14.8 ± 1.5 h LT, i.e., aligned with the trailing region of the ENA enhancement as shown by the red curve in Figure 3c, rather than the location of the most intense ENA fluxes at ∼19 h LT. It is also worth noting that the ENA intensity does not sharply drop off at the trailing edge of the enhancement, rather broadly decreases in intensity from ∼18 h to ∼13 h LT, i.e., roughly over the azimuthal width of the auroral form. We also note that the location of the double feature highlighted in the orange box in Figure 1h extrapolated to the time of the ENA image is essentially identical to that of the peak of the wider form. Unfortunately, the low latitude of the ENA observations, and the resulting uncertainty in the ENA intensity gradients, precludes an estimation of the brightness of the resulting auroral emissions, and the auroral intensity was fading during the observation interval such that it would not be expected to be very high by the time of the ENA image if the fading continued at the observed rate. Nevertheless, it is our conjecture that the auroral emission is associated with the upward field-aligned continuity current associated with the trailing region of the eastward directed partial ring current arising from the heated plasma manifest later in the ENA image. While there may well be a diffuse auroral component driven by precipitating hot plasma associated with the ENA emissions, this does not seem to be the origin of the bulk of the emission observed in this data set. This may also be the case for the patchy emission equatorward of the bursts in the day 95 images. We note that this situation differs somewhat from that discussed by *Mitchell et al.* [2009], who showed that patchy auroral emission near ∼20°colatitude observed by Cassini UVIS was coincident with enhanced ENA emissions between ∼10 and 20 *R*_*S*_. Those auroral emissions were identified with filamentary field-aligned currents associated with the partial ring current, although it is worth noting that their latitude was well equatorward of those considered here.

## 5. Summary

We have presented images of Saturn's northern UV auroras obtained with HST in April/May 2013. We have shown that two auroral storms were observed during the interval, which exhibited dynamics not hitherto reported. Three bursts of auroral emission were observed ∼10–15 min apart at the poleward boundary of an auroral storm, propagating at ∼4.1 km s^−1^, corresponding to ∼330% rigid corotation, from near ∼01 h LT toward ∼08 h LT. We have suggested that these features resemble terrestrial PBIs and are indicative of ongoing, bursty reconnection of lobe flux in the magnetotail, whose onset propagates across the tail toward the dawn flank at speeds of ∼1340 km s^−1^. This provides strong evidence that Saturn's auroral storms result from large-scale flux closure induced by solar wind compressions as suggested by *Cowley et al.* [2005]. We also discuss an auroral event observed on day 140, which likely corresponds to the later evolution of a similar solar wind-induced storm, and track the evolution of the form over three HST orbits. We show that the emission rotates at ∼45% rigid corotation. Extrapolating the rotation to the time of an ENA observation places the auroral emission at the trailing region of the ENA enhancement and is thus conjectured to result from the upward field-aligned continuity current flowing into the associated partial ring current in this region. We suggest that this then accounts for the main body of auroral storm emissions.

## Key Points

- Saturn's auroral storms exhibit fast-propagating bursts on the poleward boundary
- These are similar to terrestrial PBIs, indicating ongoing closure of lobe flux
- Subsequent emission maps to the trailing Region 2 current of an ENA enhancement

## Supporting Information

Readme
