# Supplementary material for: Dynamic auroral storms on Saturn as observed by the Hubble Space Telescope
Source: Geophys Res Lett. 2014 May 20;41(10):3323–30. doi: 10.1002/2014GL060186 (PMC4459195; doi:10.1002/2014GL060186)
Supplement: Readme [file grl0041-3323-sd1.pdf]

Auxiliary Material for manuscript 2014GL059373

Dynamic auroral storms on Saturn as observed by the Hubble Space Telescope.

J. D. Nichols(1), S. V. Badman(1,2), K. H. Baines(3), R. H. Brown(4), E. J. Bunce(1), J. T. Clarke(5), S. W. H. Cowley(1), F. J. Crary(6), M. K. Dougherty(7), J.-C. Gerard(8), A. Grocott(1,2), D. Grodent(8), W. S. Kurth(9), H. Melin(1), D. G. Mitchell(10), W. R. Pryor(11), T. S. Stallard(1)

(1) Department of Physics and Astronomy, University of Leicester, University Road, Leicester, LE1 7RH, UK

(2) Department of Physics, Lancaster University, Lancaster, LA1 4YB, UK

(3) Space Science and Engineering Center, University of Wisconsin-Madison, 1225 W. Dayton St., Madison, WI 53706, USA

(4) Lunar and Planetary Lab, University of Arizona, Tucson, AZ 85721, USA

(5) Center for Space Physics, Boston University, 725 Commonwealth Avenue, Boston, MA 02215, USA

(6) Laboratory for Atmospheric and Space Physics, University of Colorado, 1234 Innovation Dr., Boulder, CO 80303, USA

(7) Blackett Laboratory, Imperial College London, London, SW7 2AZ, UK

(8) Laboratoire de Physique Atmospherique et Planetaire, B5c, Universite de Liege, Allee du 6 Aout 17, B-4000 Liege, Belgium

(9) Department of Physics and Astronomy, University of Iowa, Iowa City, IA 52241, USA

(10) Applied Physics Laboratory, Johns Hopkins University, 11100 Johns Hopkins Road, Laurel MD 21042, USA

(11) Central Arizona College, 8470 N. Overfield Road, Coolidge AZ 85228, USA

Geophysical Research Letters, 2014

Introduction

This file contains a movie of all the HST images considered in this study, i.e. those obtained on days 95 and 140.

1. 2014gl060186-ms01.m4v This file contains a movie of all the HST images considered in this study, i.e. those obtained on days 95 and 140. The format is as described in the paper.
